# Impact of Obesity on Lumbar Puncture Outcomes in Adults with Acute Lymphoblastic Leukemia and Lymphoma: Experience at an Academic Reference Center

**Published:** 2019-07-01

**Authors:** José Carlos Jaime-Pérez, Guillermo Sotomayor-Duque, Patrizia Aguilar-Calderón, Lorena Salazar-Cavazos, David Gómez-Almaguer

**Affiliations:** Department of Hematology, Internal Medicine Division, Dr. José E. González University Hospital, School of Medicine, Universidad Autónoma de Nuevo León, Monterrey, Mexico

**Keywords:** Acute lymphoblastic leukemia, Central nervous system (CNS) relapse, Lumbar puncture, Obesity, Traumatic lumbar puncture

## Abstract

**Background: **Lumbar puncture (LP) is a hematology procedure that can require repeated attempts leading to traumatic LP (TLP), which has been related to the central nervous system (CNS) relapse. LP success can depend on the size and anatomy of the patient and the skill of the hematologist. The main objective was to determine the influence of body mass index (BMI) on LP outcomes.

**Materials and Methods: **Adults with lymphoid malignancies requiring LP were included prospectively over one year; hematology residents performed most procedures. A 22-gauge Quincke needle was employed. Comparison between non-traumatic vs. traumatic LPs according to BMI, CNS relapse, and residents’ year was performed.

**Results:** Fifty-four patients with a mean age of 31.5±15.57 years were included. Diagnosis was Acute Lymphoblastic Leukemia-B (74%), Acute Lymphoblastic Leukemia-T (13%) and Non-Hodgkin Lymphoma (13%). 227 LPs were performed, 121 (53.3%) successful, 98 (43.2%) traumatic, 11 (11.2%) TLPs were macroscopically detectable and 87 (88%) microscopic; 8 (3.5%) were dry-taps. Median time between punctures was 11 days (1-202). Median BMI was 25 (22.8-39.6). Main indication for LP was prophylactic (74.5%); 39.2% were performed by first-year, 35.2% by second-year, 19.6% by third-year hematology residents. No difference (p = 0.145) for a TLP was found among residents. A BMI ≥30 (p = 0.040), non-palpable intervertebral space (p = 0.001) and more than one attempt (p = 0.001) were significant for TLP. TLP was not associated with CNS relapse (p = 0.962).

**Conclusion: **Obesity predicted a TLP. A traumatic puncture did not increase the risk of CNS relapse at one-year follow-up.

## Introduction

 Central nervous system (CNS) affection at diagnosis is a complication in 5-10% of adult leukemias and lymphomas^   1 ^. Risk factors for this complication at diagnosis include high serum lactic dehydrogenase, T-ALL, B-mature ALL, and a leukocyte count > 30,000μL^   2 ^. Thus, in patients with these malignancies, lumbar puncture (LP) is necessary to know the status of the disease as well as to initiate intrathecal therapy^3^.

Performance of a LP is influenced by several factors, including patients’ weight and BMI. The distance between the subarachnoid space and the skin is directly influenced by the BMI. Most reports studying LP outcomes in adults have been conducted on patients with neurologic diseases. In one study, the distance in adults was measured by magnetic resonance imaging; the median was 6.2 cm with a median BMI of 29.8. It was found that the distance increases as the BMI does, which causes greater difficulty for the clinician to perform the procedure^4^. In a retrospective analysis at a neurology center, the factors associated with a successful LP were assessed. BMI was the most important factor, with greater difficulty in patients having a BMI >34^   5 ^. Experience with adults in a dedicated LP clinic recently documented high BMI, female sex and older age as factors associated to an unsuccessful LP, whereas younger age, female sex and hypertension were associated to post-lumbar puncture headache^   6 ^. In another report including 52 adult patients, an abdominal circumference greater than 94 cm or a BMI greater than 26 were associated with greater difficulty in the identification of the intervertebral space and in performing a LP^   7 ^. Overweight and age were also identified as predictors of greater difficulty when carrying out LP in patients with a BMI >30 and age >65 years^   8 ^. Importantly, in the United States 34.9% of people suffer from obesity ^   9 ^, whereas in Mexico the prevalence of obesity is 39.7%, and other 29% of the adult population are overweight ^   10 ^. 

Although LP is a routine procedure in hematology, it is sometimes difficult to carry out, requiring repeated attempts, even when performed by experienced physicians^   11 ^**.** ALL and some lymphomas require periodic LP during treatment   ^12^^,^^13^, and increased risk of CNS relapse in adults with ALL sustaining a traumatic lumbar puncture (TLP) has been reported^14^.

There is no standardized definition of what constitutes a difficult LP; no prospective studies specifically describing the number of punctures required to obtain craniospinal fluid (CSF) and the influence of years of residency training on LP outcome in adult hematology patients are available. Remarkably, use of routine ultrasound to guide LP was not found useful in adults^   15 ^.

The influence of BMI and years of hematology residency training on LP results and TLP rate in an academic reference center were documented over a year.

## MATERIALS AND METHODS

 A prospective, longitudinal, descriptive non-experimental study of patients attending the Hematology Department, Internal Medicine Division of the “Dr. José E Gonzalez” University Hospital and School of Medicine of the Universidad Autónoma de Nuevo Leon in Monterrey, Mexico was carried out. Patients of both sexes, > 16 years of age, with lymphoid malignant hematological disease and an indication for LP during the period from November 1, 2015 to October 31, 2016 were included and followed up for one year. The Institutional Review Board and Ethics Committee approved the protocol for the study.

Before the procedure, the reasons for LP and details of the technique were explained to all participants. Moreover, signed informed consent was obtained from all individual participants included in the study.

Following local anesthesia, LP with a 22-gauge Quincke needle was performed. Once the needle crossed the yellow ligament and was placed inside the subarachnoid space, ≥3.0 mL of CSF was obtained. CSF was immediately sent to the hematology laboratory for its study. LP findings were recorded in an *ad hoc* format including year of training of the resident performing the procedure. LP was considered successful when uncontaminated CSF was obtained at first or second attempt; failed when no CSF could be drawn (dry tap); TLP was defined by CSF visually contaminated with RBCs or if ≥ 10 erythrocytes/L were found under microscopic examination^   16 ^. We considered a LP difficult if two consecutive intents failed and a change in intervertebral space was necessary. Patients who did not accept the procedure were excluded from the study.

Demographic data were collected, and weight and height were determined to calculate the BMI by the Mosteller formula^   17 ^. LP was carried out by hematology residents and hematology professors. Entrance to our program requires 4-year internal medicine board-certified training.

The hematologic profile before LP was verified and apheresis platelets transfused before the procedure when the platelet count was <50,000/μL ^18^ . The total number of attempts and success or failure for each LP was registered and entered in a database. In addition, characteristics of the CSF and findings under microscopic observation were documented. Adverse events were recorded during or after each procedure following the common terminology criteria for adverse events, CTCAE ^   19 ^. 

**Statistical analysis**

For analysis, the SPSS statistical package v. 22.0 (IBM SPSS Statistics software, IBM Corp., Armonk, NY) was used. Descriptive analysis consisted of medians with ranges. For the quantitative variables, comparison between groups with and without traumatic LP was made by means of Student's t-test or the Mann-Whitney U test. Risk factors for relapse were analyzed by the Cox regression method with a 95% CI. A value of P<0.05 was considered significant.

## Results

 A total of 54 patients, 33 men (61%) and 21 women (39%) with a mean age of 31.5±15.57 years were included in the study. They were followed during one-year. The most frequent diagnosis was ALL B (74%), followed by ALL-T and NHL (13% each). Median BMI was 25(22.8-39.6); 51.9% of the patients presented a BMI > 25, Table 1. Chemotherapy regimens were administered according to standardized protocols; the results at our center have been published ^20^^,^^21^ . Additional demographic data, parameters of the complete blood count and CSF characteristics are shown in Tables 1 and 2. 

**Table 1:** Important characteristics of 54 adults with acute lymphoblastic leukemia (n=47) or non-Hodgkin lymphoma (n=7) at the time of lumbar puncture

**Patient characteristics**	**N (%)**
Age mean with SD	31.5±15.57
Sex	
Female	21 (39)
Male	33 (61)
Diagnosis	
ALL-B	40 (74)
ALL-T	7 (13)
NHL	7 (13)
Weight, median (range), kg	66.8 (50-140)
Height, median (range), m	1.64 (1.48-1.88)
BMI, median (range), kg/m^2^	25 (22.8-39.6)
<18.5 Low, n (%)	6 (11.1)
18.5-24.9 Normal, n (%)	20 (37.03)
25-29.9 Overweight, n (%)	15 (27.77)
≥30 Obesity, n (%)	13 (24.1)
Intervertebral space not palpable, n (%)	13 (23)

SD, Standard Deviation, ALL-B, B-cell acute lymphoblastic leukemia; ALL-T, T-cell acute lymphoblastic leukemia; NHL, non-Hodgkin lymphoma; BMI, body mass index

**Table 2:** Salient craniospinal fluid (CSF) and complete blood count (CBC) laboratory results for 54 adults with acute lymphoblastic leukemia and non-Hodgkin lymphoma undergoing lumbar puncture (n=219)

**Craniospinal fluid (CSF) findings**	**Median (range)**
White blood cells/µL	0 (0-3102)
Erythrocytes/µL	10 (0-1900)
CNS infiltration, n (%)	21 (9.6)
Macroscopic TLP, n (%)	11 (11.2)
Microscopic TLP, n (%)	87 (88)
Complete blood count (CBC)	Median (range)
Leucocytes, x10^3^/µL	3165 (2.2-247,900)
Hemoglobin, g/dL	10.5 (4.5-14.9)
Platelets, x10^3^/µL	160 (90-661)
Blasts, %	68.5 (8-100)
Philadelphia chromosome, n (%)	9 (16.6%)
Serum LDH (IU/L)	612.6 (120-5,000)

CNS, central nervous system; LDH lactate dehydrogenase

**Procedures**

There were 227 LP, of which 121 (53.3%) were successful, 98 (43.2%) traumatic and 8 (3.5%) dry taps. Eleven (11.2%) TLPs were macroscopic (visually detected) vs. 87 (88.8%) microscopic. The median time between punctures was 11 days (1-202) and the number of attempts was 1 (1-5). Main indication for the puncture was prophylactic (74.5 %). First-year hematology residents performed 39.2%, second year 35.2% and third-year 19.6% LP. A professor performed the procedure in 6%, and these were considered difficult LP, requiring a change in intervertebral space. Salient aspects of the data are shown in Table 3.

**Table 3:** Lumbar puncture (LP) procedures (n=227) and their characteristics in 54 adults with acute lymphoblastic leukemia and non-Hodgkin lymphoma

Lumbar puncture characteristic	Variable, (%)
**Total LP**	227
**Successful, n (%)**	121(53.3)
**Traumatic, n (%)**	98 (43.2)
**Failed, n (%)**	8 (3.5)
**Prophylactic (%)**	169 (74.5)
**Therapeutic (%)**	58 (25.5)
**Time between lumbar punctures, ** **median, days (range)**	11 (1-202)
**Attempts, median (range)**	1 (1-5)
**More than one physician doing ** **the procedure, n (%)**	27(11.8)
**Hematology resident academic ** **year, n, (%)**	
**First year**	89 (39.2)
**Second year**	80 (35.2)
**Third year**	44 (19.6)
**Professor**	14 (6.0)

**Variables associated to traumatic lumbar puncture**

Of all LP in patients with a BMI ≥30, 59.7% were traumatic vs. 40.3% in those with a BMI <30, (p = 0.04). Patients in whom the intervertebral space was difficult to locate or was not palpable suffered a TLP in 70% vs. 35% in those with a palpable space (p = 0.001). The need for more than one attempt to perform the procedure was associated with a TLP in 66.6% vs. 35.8% of the procedures when a single attempt was needed (p = 0.001). Platelet count before LP and time between punctures were not statistically significant for a TLP (p= 0.640). Also, no significant association was found between academic year of the hematology resident performing the procedure and a traumatic puncture (p = 0.145), Table 4. In 71.7% of patients with a BMI ≥30 the intervertebral space was not easily palpable (p = 0.001), whereas 95% of patients with an easily palpable space required a single attempt to obtain CSF (p = 0.001), Table 5.

**Table 5:** Influence of body mass index (BMI) on palpable intervertebral space and comparison between attempts vs. palpable/non-palpable intervertebral space in adults with lymphoid malignancy

**Variable**	**Palpable space,** **N (%)**	**Notpalpable space, ** **N (%)**	**P**
BMI **<** 30	151 (95)	17 (28.3)	0.001
BMI **>** 30	8 (5)	43 (71.7)	
1 attempt	127 (80)	29 (48)	0.001
**>**1 attempt	32 (20)	31 (52)	

**Complications**

Among 227 procedures, 27 (12%) had some clinical complication, the most frequent being post-puncture headache in 19 (8.4%) and local pain in 5 (2.2%). CNS toxicity occurred in 2 patients (0.9%) associated to methotrexate; there was one episode of transient hypotension (0.4%). In patients who suffered a TLP, 15 complications developed vs.12 in those in which LP was uncomplicated (p = 0.308), Table 4.

**Table 4:** Influence of body mass index (BMI) < or > 30, palpable vs. non-palpable intervertebral space, complications and central nervous system (CNS) infiltration according to traumatic (TLP) vs. non-traumatic lumbar puncture (Non-TLP) in 54 adults with acute lymphoblastic leukemia (n=47) and lymphoma (n=7)

**Variable**	**TLP, n (%)**	**Non-TLP, n (%)**	**p**
BMI <30, 24.4 (22.8-29.76)	67 (68.3)	100 (82.6)	0.040
BMI >30, 30.48 (30.1-39.6)	31 (31.6)	21 (17.4)	
Platelets> 50000/µL	91 (92.9)	110 (90.9)	0.640
Platelets< 50000/µL	7 (7.1)	11 (9.09)	
Palpable intervertebral space	56 (57.1)	103 (85.1)	0.001
Non-palpable intervertebral space	42 (42.9)	18 (14.9)	
Time between procedures<15 days	54 (55.1)	67 (54)	0.996
Time between procedures >15 days	44 (44.9)	54 (53.5)	
1 attempt	56 (57.1)	100 (82.7)	0.001
>1 attempt	42 (42.9)	21 (17.3)	
First-year resident	41 (42)	40 (40)	0.145
Second-third-year resident	50 (52)	74 (60)	
Complications			0.308
Yes	15 (15)	12 (10)	
No	83 (85)	109 (90)	
CNS Infiltration			0.944
Yes	11 (11)	10 (8)	
No	87 (89)	111 (92)	

**Central Nervous System involvement**

At first-time procedure, eleven LP (11%) demonstrating CNS infiltration were found in 98 TLP vs. 10 (8%) in 121 non-traumatic punctures (p =0.944), Table 4. No statistical difference at a median follow-up of 12 months was found in CNS relapse rates when patients having a TLP were compared to those with a non-traumatic procedure (p = 0.962). Eight LP were not assessable as no CSF was drawn. Comparison of CNS relapse between TLP with and without blasts during one-year follow-up by Cox-regression univariate analysis was not significant (p=0.689). Comparison of relapse to the CNS in patients having macroscopic (n=4) vs. microscopic (n=5) TLP during one-year follow-up was not significant (p=0.190).

## Discussion

 Lumbar puncture is frequently performed during treatment of patients with ALL and lymphoma; thus, it is a critical component of therapy and hematology training. There are few studies assessing the impact of BMI on the success or failure of LP in adult patients. Since the prevalence of overweight and obesity has increased over the last decades and more so in low-middle income countries like ours, we investigated how BMI influences LP outcome. In addition, assessment of CNS relapse after TLP in adults is scarcely documented, and prospective data on LP performance by hematology residents is lacking.

Although LP is generally a safe procedure, there are associated complications such as post-puncture headache, lower limb pain, traumatic puncture, local infection at the puncture site, and less common ones such as meningitis, subarachnoid hemorrhage or cerebral herniation^   22 ^. In a study, a post-puncture headache frequency of 37% was found, with a higher prevalence in women; it was also documented that its duration was longer in patients with a higher BMI^   23 ^. We confirmed that headache was the most frequent complication of LP, although at a lower incidence of 8% in our patients; bed rest plus moderate analgesia sufficed to alleviate this symptom in all our cases. A report found 8-hour bed rest after intrathecal chemotherapy to be optimal for minimizing this complication^   24 ^. Another study found intrathecal isotonic saline injection after LP to be effective for prevention of headache^   25 ^. Also, atraumatic LP needles have recently been found to offer a reasonable benefit in reducing headache^   26 ^.

Infiltration of the CNS in NHL is significantly lower than observed in ALL, 4-5%; in this respect, we recently reported an 8.5% frequency of initial CNS involvement in 94 adults with ALL^   20 ^. On the other hand, CNS infiltration in NHL is related to bulky disease and histological subtype with diffuse, lymphoblastic, blast variant mantle and Burkitt's lymphoma being more affected and in these cases prophylaxis is recommended^   2 ^. We performed LP in seven patients with NHL, none revealed infiltration at diagnosis or during the one-year follow up.

Of the 227 procedures performed in the year of the study, 98 TLPs (43.2%) were recorded, of which only 11 (11.2%) were macroscopically detectable. A TLP frequency of 6.7-69.6% has been previously reported in adults with ALL^14^^,^^18^ , whereas in children this rate varies from 0.8% in India^   27 ^, 13.9% in the United States^16^, 17.9% in Canada^   28 ^, and 24.7% in Brazil^   29 ^. 

The high prevalence of overweight/obesity in our population adding up to 70% was the main factor contributing to the elevated TLP rate observed; the fact that ours is a reference centre caring for an elevated proportion of adults with high-risk ALL requiring performing more LPs also appears to have influenced our results. Interestingly, we have recently documented a higher incidence of ALL than acute myeloid leukemia in adults in Mexico ^30^^,^^31^ , which leads to the need for performing a higher number of LP procedures. 

In some studies, a platelet count <50x10^9^/L, time between punctures < 15 days, and lower academic degree of the physician are described as factors for a TLP^   18 ^. In agreement with a recent publication, these variables did not reach significance in our study^32^. A Cochrane systematic review found that there was no evidence for a difference in the risk of minor bleeding in participants who received platelet transfusions and those who did not before a lumbar puncture^   33 ^.

Interestingly, although first and second-year residents performed most of the procedures, no statistical association for a TLP according to the year of hematology training was found, reflecting that some factors for a TLP are not operator-dependent, and thus potentially modifiable, including use of atraumatic LP needles instead of Quincke beveled-type. 

Although the mechanisms for bleeding within the CSF at the time of LP are not known with certainty, an over-insertion of the needle that causes laceration of the internal vertebral venous plexuses in the epidural space is referred to as a conspicuous cause ^13^^,^^34^ . Sometimes, when the location of the needle is appropriate, there is contamination by erythrocytes of blood vessels surrounding nerve roots or, when the patient moves during the procedure, bleeding may come from soft tissues^35^. The presence of blood in the CSF alters the cell count, increases the level of proteins and can cause false positive results in cultures or cytology, with the consequent confusion in the interpretation of the results. 

Importantly, almost 90% of our TLPs were microscopic which did not increase the risk of CNS relapse at one-year of follow-up in our group. This contrasts with studies in children with ALL in whom contamination of CSF with circulating leukemic blasts during diagnostic LP adversely affected treatment outcome and was an indication to intensify intrathecal therapy^   16 ^.

This is the first prospective study, documenting LP outcomes in adults with lymphoid malignancies and also the first distinguishing between macroscopic and microscopic TLP and its influence on CNS relapse; importantly, the efficiency of hematologists in training for performing the procedure was also assessed.

## CONCLUSION

 Several conclusions can be drawn from these results: Obesity and difficulty to locate the intervertebral space predicted a TLP; a traumatic LP was not associated with academic year of the hematology resident performing the procedure and appears to result from the elevated proportion of overweight and obesity; TLP did not increase the risk of CNS relapse after one year of follow-up in our population; after prophylactic platelet transfusion thrombocytopenia was not a risk factor for TLP. 

Studies in populations with different BMI distribution are necessary to better define the role of obesity in LP outcomes in hematology patients.
